# Efficacy and safety of zuberitamab in the treatment of primary membranous nephropathy: an observational study

**DOI:** 10.3389/fimmu.2025.1741564

**Published:** 2026-01-12

**Authors:** Li Zong, Sha Wang, Ke Zhao, Fang-yi Lv, Shan-kui Qian, Xiang-dong Yang

**Affiliations:** Nephrology Department, Qilu Hospital of Shandong University, Jinan, Shandong, China

**Keywords:** anti-PLA2R antibody, membranous nephropathy, remission, rituximab, zuberitamab

## Abstract

**Research Background:**

B lymphocyte-mediated adaptive immunity is pivotal in the pathogenesis of membranous nephropathy (MN). This study evaluated the efficacy and safety of zuberitamab, a novel anti-CD20 monoclonal antibody, in primary membranous nephropathy (PMN).

**Research Methods:**

We retrospectively analyzed 25 patients with PMN treated with zuberitamab (zuberitamab group). Using 1:1 propensity score matching, we selected a comparable control cohort of 25 PMN patients who received rituximab during the same period (RTX group). The primary endpoint was the 12-month overall remission rate (CR + PR).

**Research Results:**

All 25 patients (100.0%) in the zuberitamab group achieved clinical remission (CR 80.0%) at 12-month, with 11 patients achieving complete immunological remission. Compared to the RTX group, the complete remission (CR) rate was significantly higher (80.0% vs 32.0%, P<0.01). Kaplan-Meier analysis demonstrated a higher complete remission rate (log-rank P<0.001; HR = 4.187, 95% CI [2.285-7.672]). Zuberitamab induced progressive urine protein-to-creatinine ratio (uPCR) reduction (all P<0.001) and sustained albumin improvement (3-month: P<0.05; 6/9/12-month: P<0.001). Peripheral blood CD19^+^ B-cell counts decreased to < 5 cells/μl at 6-month among the 22 patients with complete follow-up data, and remained consistently low throughout follow-up. No severe adverse events were observed in either group.

**Conclusion:**

Zuberitamab may be a promising treatment option for PMN, demonstrating a high complete remission rate and sustained proteinuria reduction in this study. Large-scale prospective studies are warranted to confirm these findings.

## 1 Introduction

Membranous nephropathy (MN) represents a pathological entity characterized by subepithelial immune complex deposition along the glomerular basement membrane (GBM), accompanied by diffuse GBM thickening. Primary membranous nephropathy (PMN), accounting for approximately 80% of cases and alternatively termed idiopathic membranous nephropathy (IMN), remains etiologically undefined (1). As a leading global cause of adult nephrotic syndrome (20-37% of cases) (2), PMN ranks as the second or third most common etiology of end-stage renal disease among primary glomerulonephritides (3). Without timely intervention, progressive renal dysfunction occurs in approximately two-thirds of MN patients within a 10-year period (4).

Current research generally suggests that B lymphocyte-mediated adaptive immunity plays a key role in the pathogenesis of MN (5). Anti-CD20 monoclonal antibodies, such as rituximab (RTX), exert therapeutic effects in glomerular diseases through CD20 binding and subsequent B-cell depletion (6). RTX monotherapy can induce clinical remission in up to 80.2% of treatment-naive and relapsed PMN patients and clear anti-PLA_2_R antibodies (7). The GEMRITUX trial revealed superior outcomes when combining RTX with non-immunosuppressive antiproteinuric treatment (NIAT), achieving significantly higher remission rates than NIAT alone (64.9% vs 34.2%, P<0.01) after 17-month median follow-up, without additional safety concerns (8). The MENTOR trial reported markedly higher 24-month remission rates compared to cyclosporine (60% vs 20%, P<0.001) (9). These robust efficacy and safety profiles have established RTX as a first-line therapy for PMN (10). Nevertheless, more than one-third of MN patients exhibit inadequate response to RTX (9, 11), underscoring the necessity for developing more effective alternative treatments.

Zuberitamab (HS006) is a novel human-mouse chimeric anti-CD20 monoclonal antibody that mediates B-cell depletion primarily through antibody-dependent cellular cytotoxicity (ADCC). It shares therapeutic indications with RTX, including CD20-positive diffuse large B-cell lymphoma (DLBCL) (12). Structural analyses indicate that zuberitamab binds to distinct epitopes on the CD20 antigen compared to RTX, which is associated with differences in binding affinity (13, 14). Following clinical evaluation, zuberitamab received marketing authorization from China’s National Medical Products Administration (NMPA) in May 2023 and was subsequently included in the national medical insurance formulary in December 2023.This study employed a retrospective, single-center, real-world design to evaluate the therapeutic efficacy and safety of zuberitamab in treating PMN. The investigation aimed to generate clinically relevant evidence that could expand treatment accessibility for PMN patients.

## 2 Methods

### 2.1 Participants

This retrospective single-center cohort study included 25 MN patients treated at the Department of Nephrology, Qilu Hospital of Shandong University, from April 2023 to April 2024. Inclusion criteria: (1) Adults aged 18–80 years; (2) Biopsy-proven MN or serum anti-PLA_2_R antibody titer ≥14 RU/mL by ELISA; (3) Completion of ≥ 1 zuberitamab treatment cycle (mono- or combination therapy); (4) Follow-up time of no less than 12 months. Exclusion criteria: (1) Concurrent experimental drug use; (2) Uncontrolled conditions causing acute renal impairment; (3) Active malignancies; (4) Acute or chronic infections; (5) Comorbid autoimmune disorders; (6) Known drug hypersensitivity; (7) Pregnancy or lactation. This study has been approved by the Ethics Committee of Qilu Hospital of Shandong University.

Propensity scores were generated using a logistic regression model that included the following covariates: age, sex, baseline uPCR, baseline eGFR and baseline serum albumin level. One-to-one matching was performed using the nearest-neighbor algorithm without replacement, with a caliper width set to 0.2 of the standard deviation of the logit of the propensity score. Balance between the matched groups was assessed using standardized mean differences, with all covariates achieving a balance of < 0.1 after matching, indicating adequate balance. We concurrently collected clinical data from 25 PMN patients who received at least one dose of RTX treatment during the study period. All 50 enrolled patients completed a minimum 12-month follow-up.

### 2.2 Treatment methods

All patients received zuberitamab therapy under inpatient supervision. The recommended dosage was 375 mg/m² body surface area (BSA). The specific infusion schedule (either 4×0.5g or 2×1g, both biweekly) and treatment intervals were determined through shared decision-making, incorporating established anti-CD20 protocols for PMN and individual clinical assessment. The use of concomitant immunosuppressants was at the treating physician’s discretion, based on the patient’s proteinuria severity and immunological profile. Following aseptic preparation, the calculated dose was reconstituted in 0.9% normal saline within a sterile, endotoxin-free infusion bag to achieve a final concentration of 1 mg/mL. Premedication included intravenous dexamethasone (5 mg) and intramuscular promethazine (25 mg). Infusions were initiated at 50 mg/hr, with incremental rate escalations of 50 mg/hr every 30 minutes (following an uneventful first hour) up to a maximum of 400 mg/hr, contingent upon tolerance assessment.

### 2.3 Data collection

We evaluated clinical and laboratory parameters including complete blood count, liver/kidney function, anti-PLA_2_R antibody titers, uPCR, and lymphocyte subsets at baseline (first treatment day with zuberitamab or RTX) and during follow-up visits. The data was collected retrospectively at 1, 3, 6, 9, and 12 months post-treatment. Due to financial constraints, lymphocyte count data were unavailable for six patients. Adverse events (e.g., acute infusion reactions, infections, pyrexia, cutaneous eruptions) were systematically documented and assessed by clinicians for severity and treatment-relatedness.

### 2.4 Outcomes and definitions

Clinical outcomes were the incidence of remission of proteinuria, including complete remission (CR), partial remission (PR) and nonresponse according to established criteria. The primary endpoint was the overall remission rate (CR + PR) at 12-month. CR was defined as uPCR <300 mg/g, serum albumin ≥30 g/L, and stable renal function without deterioration (15). PR was defined as uPCR between 300–3500 mg/g with ≥50% reduction from peak levels, accompanied by improved or normal serum albumin levels or stable serum creatinine (<30% increase from baseline) (15). Patients who did not meet these criteria were considered nonresponse.

Immunological outcomes were exclusively evaluated in PLA_2_R-associated MN patients. Immunological remission was defined as serum anti-PLA_2_R antibody (A-PLA_2_R) titers <14 RU/ml, measured by enzyme-linked immunosorbent assay (ELISA) (16). Immunological complete remission was defined as A-PLA_2_R <2 RU/ml (16). B cell depletion was defined as peripheral blood CD19^+^ B cell counts <5 cells/µl, monitored by flow cytometry (15).

Safety outcomes encompassed all adverse events occurring during monoclonal antibody administration and throughout the entire follow-up period. Adverse events were systematically analyzed, including incidence rates, specific nomenclature of serious adverse events, temporal occurrence patterns, and severity grading.

### 2.5 Statistical analysis

Normality of continuous variables was assessed using the Shapiro-Wilk test. Normally distributed data were expressed as mean ± standard error (SE) and analyzed using independent or paired t-tests. Non-normally distributed variables were presented as median (interquartile range [IQR]) with Mann-Whitney U or Wilcoxon signed-rank tests for comparisons. Categorical variables were reported as frequencies (percentages) and analyzed by chi-square test or Fisher’s exact test, as appropriate based on sample size and expected frequencies. Survival analysis for remission rates was performed using Kaplan-Meier curves with log-rank testing. All analyses were conducted using GraphPad Prism 8.0.2 (GraphPad Software, USA) and SPSS 25.0 (IBM Corp., USA), with statistical significance defined as two-tailed p<0.05.

## 3 Results

### 3.1 Baseline characteristics

**Figure 1:**
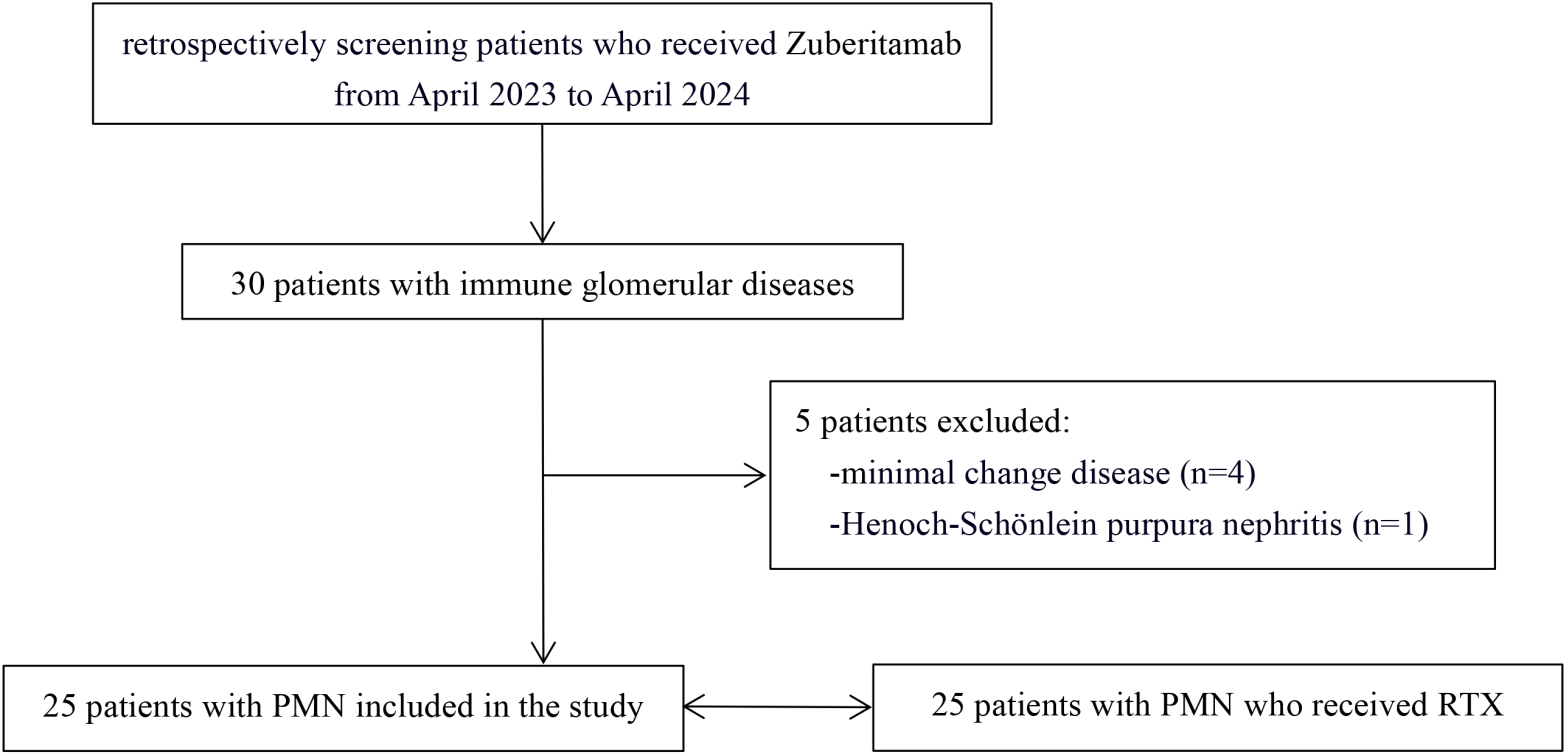
Study flow diagram.

This retrospective analysis evaluated 30 patients with immune-mediated glomerular diseases treated with zuberitamab at Qilu Hospital of Shandong University (April 2023-April 2024). After applying stringent inclusion/exclusion criteria, we excluded 5 patients for non-PMN glomerulonephritis diagnoses. The final cohort comprised 25 PMN patients (zuberitamab group). Using 1:1 propensity score matching, we selected 25 matched PMN controls treated with RTX (RTX group) during the identical observation period (**Figure 1**).

The median ages of the zuberitamab and RTX group were 59.0 (47.0-68.0) and 54.0 (43.5-60.0) years, respectively (P = 0.237). At baseline, the median uPCR was 6.8 (4.8-10.0) vs 5.6 (3.8-8.1) g/g (P = 0.286); the mean serum albumin was 28.8 ± 8.9 vs 30.7 ± 7.8 g/L (P = 0.418); the median serum creatinine was 82.0 (61.0-99.0) vs 80.0 (71.5-98.0) μmol/L (P = 0.637); the mean eGFR was 85.4 ± 32.2 vs 91.5 ± 22.7 mL/min/1.73m² (P = 0.439); the median A-PLA_2_R was 52.6 (7.7-203.9) vs 62.3 (11.9-84.2) RU/mL (P = 0.821); and the mean peripheral blood CD19^+^ B cell counts were 312.1 ± 55.6vs 330.2 ± 35.4 cells/μL (P = 0.786). Both groups contained 11 anti-PLA_2_R-positive patients. The dosing regimens between the two groups were comparable (all P>0.05) (**Table 1**).

**Table 1:** Baseline characteristics of patients with PMN included in the study.

Patients (n)	Total (n=50)	Zuberitamab (n=25)	Rituximab (n=25)	P value
Male/Female (n)	32/18	15/10	17/8	0.556
Age (years)	56.5 (46.8-62.0)	59.0 (47.0-68.0)	54.0 (43.5-60.0)	0.237
Hypertension, n (%)	31 (62.0)	17 (68.0)	14 (56.0)	0.382
Diabetes mellitus, n (%)	12 (24.0)	6 (24.0)	6 (24.0)	1.000
History (months)	11.0 (2.0-24.0)	12.0 (2.0-30.0)	10.0 (2.0-24.0)	0.938
Baseline UPCR (g/g)	6.2 (3.9-9.5)	6.8 (4.8-10.0)	5.6 (3.8-8.1)	0.286
Baseline UACR (g/g)	3.8 (2.5-6.2)	4.0 (3.2-7.8)	3.5 (2.2-5.7)	0.332
Baseline albumin (g/L)	29.8 ± 8.3	28.8 ± 8.9	30.7 ± 7.8	0.418
Baseline serum creatinine (umol/L)	81.0 (66.5-98.3)	82.0 (61.0-99.0)	80.0 (71.5-98.0)	0.637
Baseline eGFR (ml/min/1.73 m^2^)	88.4 ± 27.7	85.4 ± 32.2	91.5 ± 22.7	0.439
Baseline PLA_2_R-Ab (RU/ml)	61.7 (10.0-140.8)	52.6 (7.7-203.9)	62.3 (11.9-84.2)	0.821
Baseline CD19^+^ B-cell (RU/ml)	321.2 ± 32.4	312.1 ± 55.6	330.2 ± 35.4	0.786
Baseline neutrophil (10^9^/L)	4.0 (3.1-5.4)	4.1 (3.2-5.7)	3.9 (3.0-5.1)	0.342
Baseline leukocyte (10^9^/L)	6.9 ± 1.6	7.1 ± 1.6	6.8 ± 1.6	0.481
Baseline PCT (ng/ml)	0.047 ± 0.018	0.049 ± 0.020	0.046 ± 0.016	0.499
Baseline Hb (g/L)	134.3 ± 2.4	130.1 ± 3.7	138.5 ± 2.8	0.147
Baseline PLT (10^9^/L)	242.1 ± 6.4	246.7 ± 8.5	237.4 ± 9.5	0.485
Baseline ALT/AST	0.85 ± 0.03	0.84 ± 0.05	0.86 ± 0.05	0.697
Previous kidney biopsy, n (%)	48 (96.0)	24 (96.0)	24 (96.0)	1.000
PLA_2_R-Ab+ at the time of diagnosis,n (%)	22 (44.0)	11 (44.0)	11 (44.0)	1.000
Therapeutic schedule				
Monotherapy, n (%)	23 (46.0)	12 (48.0)	11 (44.0)	0.777
Combination therapy, n (%)	27 (54.0))	13 (52.0)	14 (56.0)	0.777
Prednisone, n (%)	9 (18.0)	4 (16.0)	5 (20.0)	1.000
Prednisone + tacrolimus, n (%)	9 (18.0)	4 (16.0)	5 (20.0)	1.000
Tacrolimus, n (%)	9 (18.0)	5 (20.0)	4 (16.0)	1.000
Zuberitamab/RTX regimen				
4×0.5g,biweekly, n (%)	28 (56.0)	15 (60.0)	13 (52.0)	0.776
2×1g,biweekly, n (%)	22 (44.0)	10 (40.0)	12 (48.0)	0.776

### 3.2 Laboratory parameter dynamics after zuberitamab treatment

**Figure 2:**
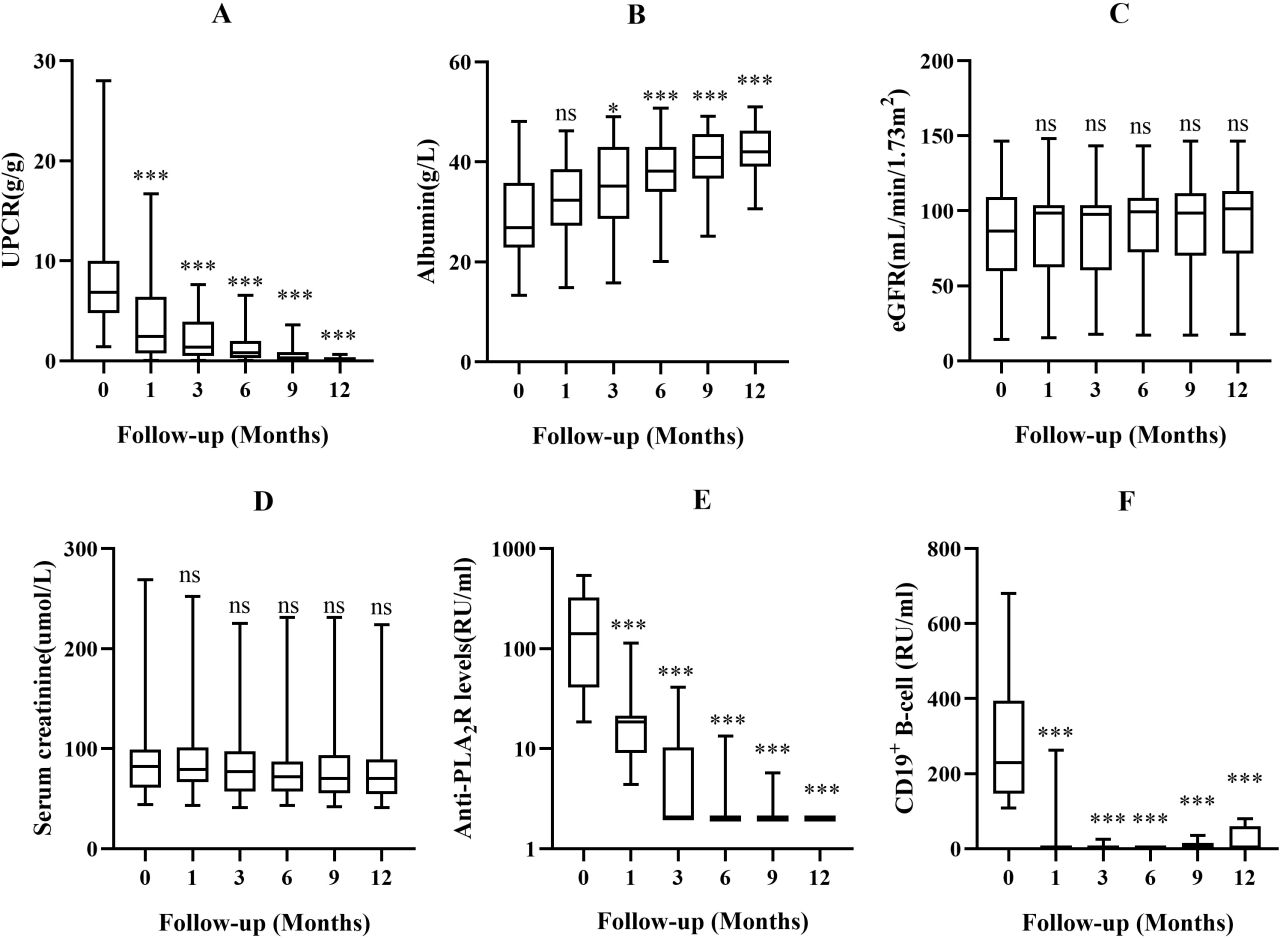
The changes in uPCR **(A)**, serum albumin **(B)**, eGFR **(C)**, serum creatinine **(D)**, A-PLA_2_R **(E)** and CD19^+^ B-cell **(F)** in the zuberitamab group. ns P> 0.05, *P< 0.05, **P< 0.01, ***P< 0.001.

Zuberitamab treatment induced progressive and sustained reductions in uPCR from month 1 onward (all P<0.001 versus baseline; **Figure 2A**). Concurrently, serum albumin levels demonstrated significant improvement beginning at month 3 (P<0.05), with progressively greater increases at subsequent timepoints (P<0.001 at 6/9/12 months; **Figure 2B**). Renal function parameters remained stable throughout follow-up, with neither serum creatinine nor eGFR showing clinically significant deviations from baseline values (**Figures 2C, D**).

Among the 11 baseline anti-PLA_2_R-positive patients, antibody titers demonstrated a progressive temporal decline: 18.6 RU/mL (IQR, 9.1-21.3) at 1 month, 2.0 RU/mL (2.0-10.3) at 3 months, 2.0 RU/mL (2.0-3.3) at 6 months, and 2.3 ± 1.1 RU/mL (mean ± SD) at 9 months, with all patients achieving complete immunological remission (titer <2 RU/mL) by 12 months. These changes demonstrated statistically significant reductions from baseline at all evaluated timepoints (all P<0.001; **Figure 2E**).

In the 22 patients with complete follow-up data, peripheral blood CD19^+^ B-cell counts exhibited rapid and sustained depletion, decreasing to 0.0 cells/μL (IQR, 0.0-2.0) by month 1 and maintaining suppression throughout follow-up (3-month: 2.0 [1.0-6.0]; 6-month: 2.0 [1.0-3.0]; 9-month: 1.0 [0.8-15.8]; 12-month: 1.0 [0.0-60.0]). All patients achieved protocol-defined depletion (<5 cells/μL) by 6 months, with counts remaining significantly below baseline at all timepoints (all P<0.001; **Figure 2F**).

### 3.3 Therapeutic efficacy: zuberitamab vs RTX

The zuberitamab group demonstrated 100.0% clinical remission (CR+PR) at 12 months, with 80.0% (20/25) achieving CR. Comparatively, the RTX group showed 96.0% (24/25) clinical remission, but only 32.0% CR. While overall remission rates did not differ significantly between groups (P = 1.000), zuberitamab yielded significantly higher complete remission rates (80.0% vs 32.0%, P<0.01; **Table 2**). Conversely, the PR rate was lower in the zuberitamab group (P<0.01), which is consistent with the shift of a greater proportion of patients achieving the higher response category (CR). It is noteworthy that, despite the favorable remission rates observed in the zuberitamab cohort, the findings should be interpreted with caution given the modest cohort size (n=25) and the single−center, observational design.

**Table 2:** Clinical and immunologic outcomes in patients.

Characteristics	Total	Zuberitamab	Rituximab	P value
Clinical outcomes				
Number of patients, n	50	25	25	-
At 6 months				
Total remission, n (%)	35 (70.0)	20 (80.0)	15 (60.0)	0.217
CR, n (%)	8 (16.0)	6 (24.0)	2 (8.0)	0.247
PR, n (%)	27 (54.0)	14 (56.0)	13 (52.0)	0.777
At 12 months				
Total remission, n (%)	49 (98.0)	25 (100.0)	24 (96.0)	1.000
CR, n (%)	28 (56.0)	20 (80.0)	8 (32.0)	0.001**
PR, n (%)	21 (42.0)	5 (20.0)	16 (64.0)	0.004**
Immunologic outcomes^a^				
Number of patients with detectable anti-PLA_2_R, n	22	11	11	-
At 6 months				
Immunologic remission, n (%)	22 (100.0)	11 (100.0)	11 (100.0)	1.000
Immunological complete remission, n (%)	17 (77.3)	9 (81.8)	8 (72.7)	1.000
At 12 months				
Immunologic remission, n (%)	22 (100.0)	11 (100.0)	11 (100.0)	1.000
Immunological complete remission, n (%)	22 (100.0)	11 (100.0)	11 (100.0)	1.000

a Only in patients with detectable anti-PLA_2_R antibody.

**P< 0.01.

**Figure 3:**
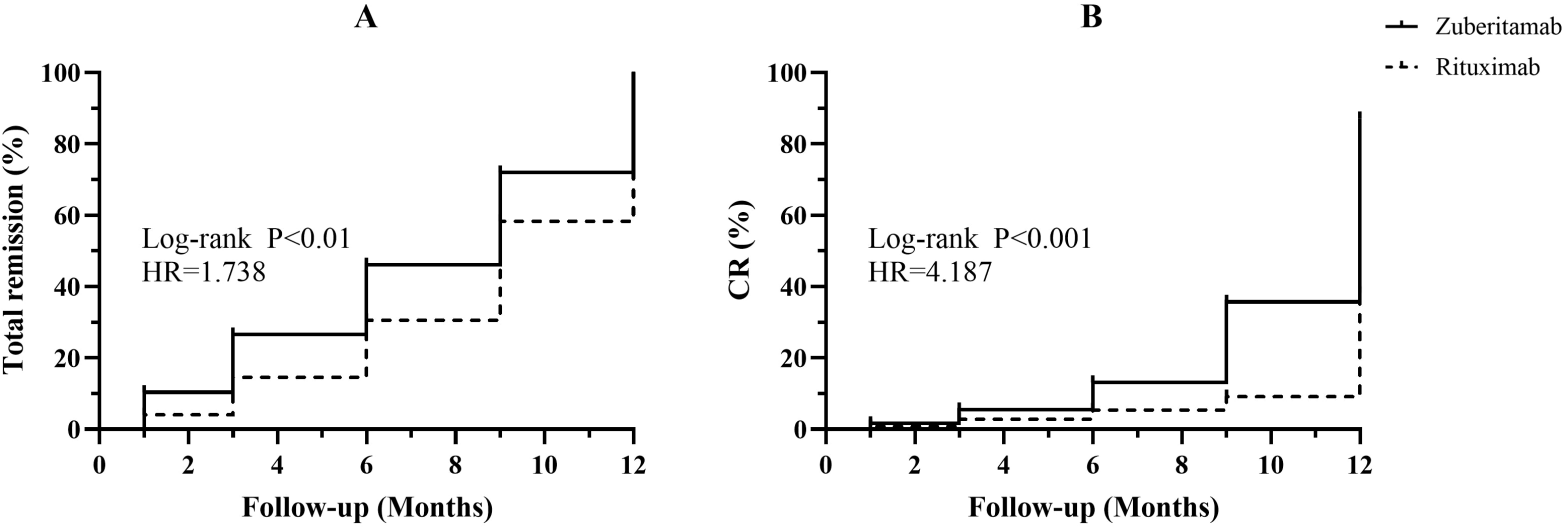
Remission of proteinuria between the zuberitamab group and rituximab group.

The Kaplan-Meier analysis revealed a significantly shorter time to overall response in the zuberitamab group compared to the RTX group (log-rank P<0.01; HR = 1.738; 95% CI [1.160-2.604]; **Figure 3A**). Furthermore, zuberitamab was associated with a higher complete response rate (log-rank P<0.001; HR = 4.187, 95% CI [2.285-7.672]; **Figure 3B**).

This study evaluated immunologic outcomes only in the 22 patients (11 in each treatment group) who were anti-PLA_2_R-positive at baseline. At 6 months, all patients in the zuberitamab group achieved immunologic remission, with 9 patients (81.8%) reaching complete immunologic remission (A-PLA_2_R < 2 RU/mL). In the RTX group, all 11 patients also achieved immunologic remission, with 8 patients (72.7%) reaching complete immunologic remission. At 12 months, all 22 patients achieved complete immunologic remission. No intergroup differences in remission rates were observed during follow-up (P = 1.000; **Table 2**).

### 3.4 Laboratory index variations: zuberitamab vs RTX

**Figure 4:**
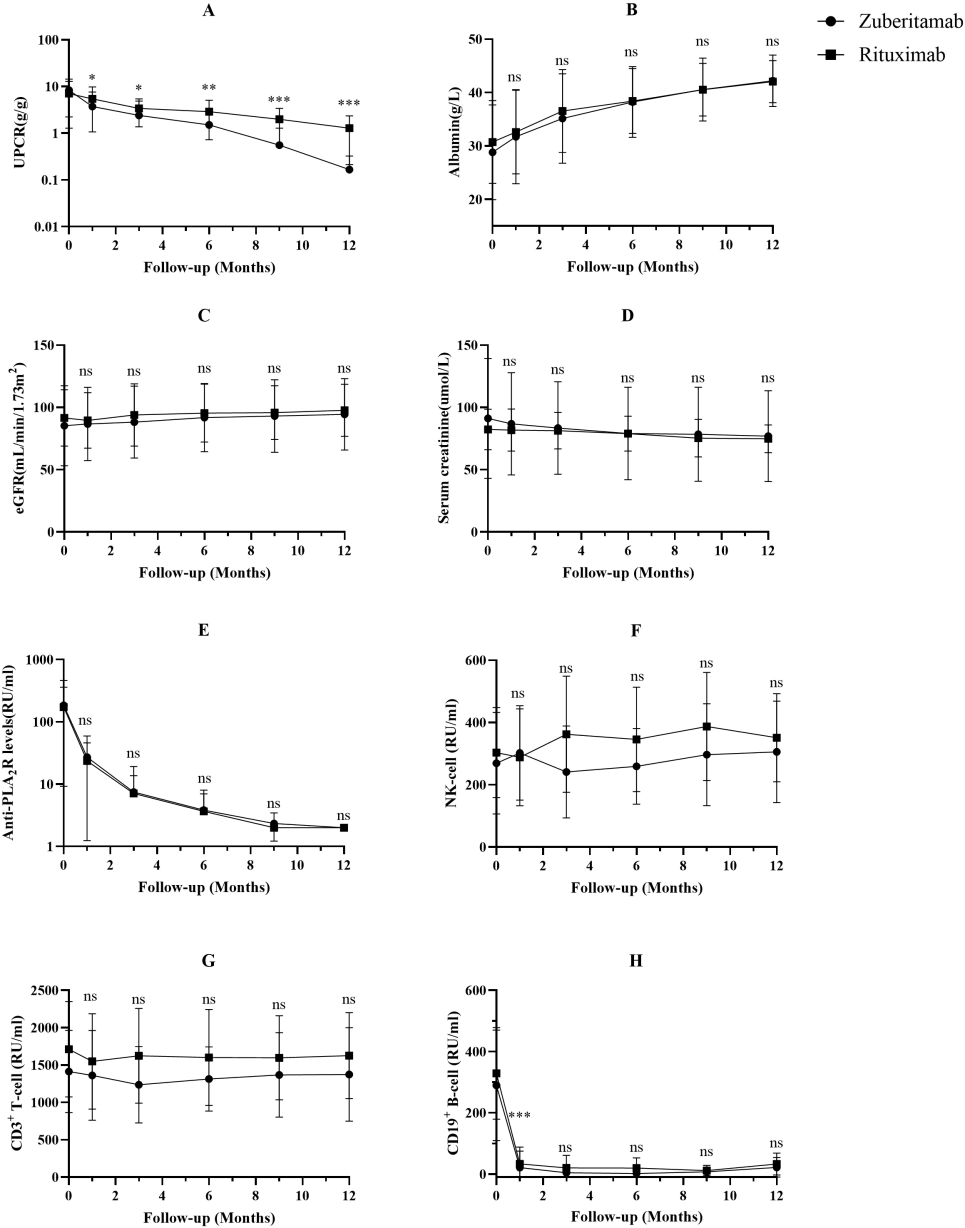
Comparison of uPCR **(A)**, serum albumin **(B)**, eGFR **(C)**, serum creatinine **(D)**, A-PLA_2_R **(E)**, NK-cell **(F)**, CD3^+^ T-cell **(G)** and CD19^+^ B-cell **(H)** between the zuberitamab group and rituximab group. ns P> 0.05, *P< 0.05, **P< 0.01, ***P< 0.001.

Throughout the follow-up, the zuberitamab group maintained significantly lower UPCR than the RTX group (all P<0.05). At month 1, median UPCR was 2.43 (0.77-6.40) g/g with zuberitamab versus 4.75 (2.83-6.85) g/g with RTX (P<0.05). The benefit widened over time. By month 6, median UPCR reached 0.82 (0.29-1.99) g/g versus 2.44 (1.60-3.66) g/g (P<0.01). At month 12, zuberitamab achieved 0.11 (0.06-0.23) g/g compared to 1.41 (0.27-1.94) g/g with RTX (P<0.001) (**Figure 4A**).

Follow-up data showed no statistically significant intergroup differences in multiple parameters: serum albumin, serum creatinine, eGFR, A-PLA_2_R titers, NK cell counts, or CD3^+^ T-cell counts (all P>0.05; **Figures 4B–G**).

Flow cytometry analysis demonstrated more profound early CD19^+^ B-cell depletion with zuberitamab versus RTX at 1 month (0.0 [0.0-2.0] vs 17.5 [3.0-53.3] cells/μL; P<0.001). During subsequent follow-up, no statistically significant differences in B-cell counts were observed between the two groups (all P>0.05; **Figure 4H**).

### 3.5 Safety

**Figure 5:**
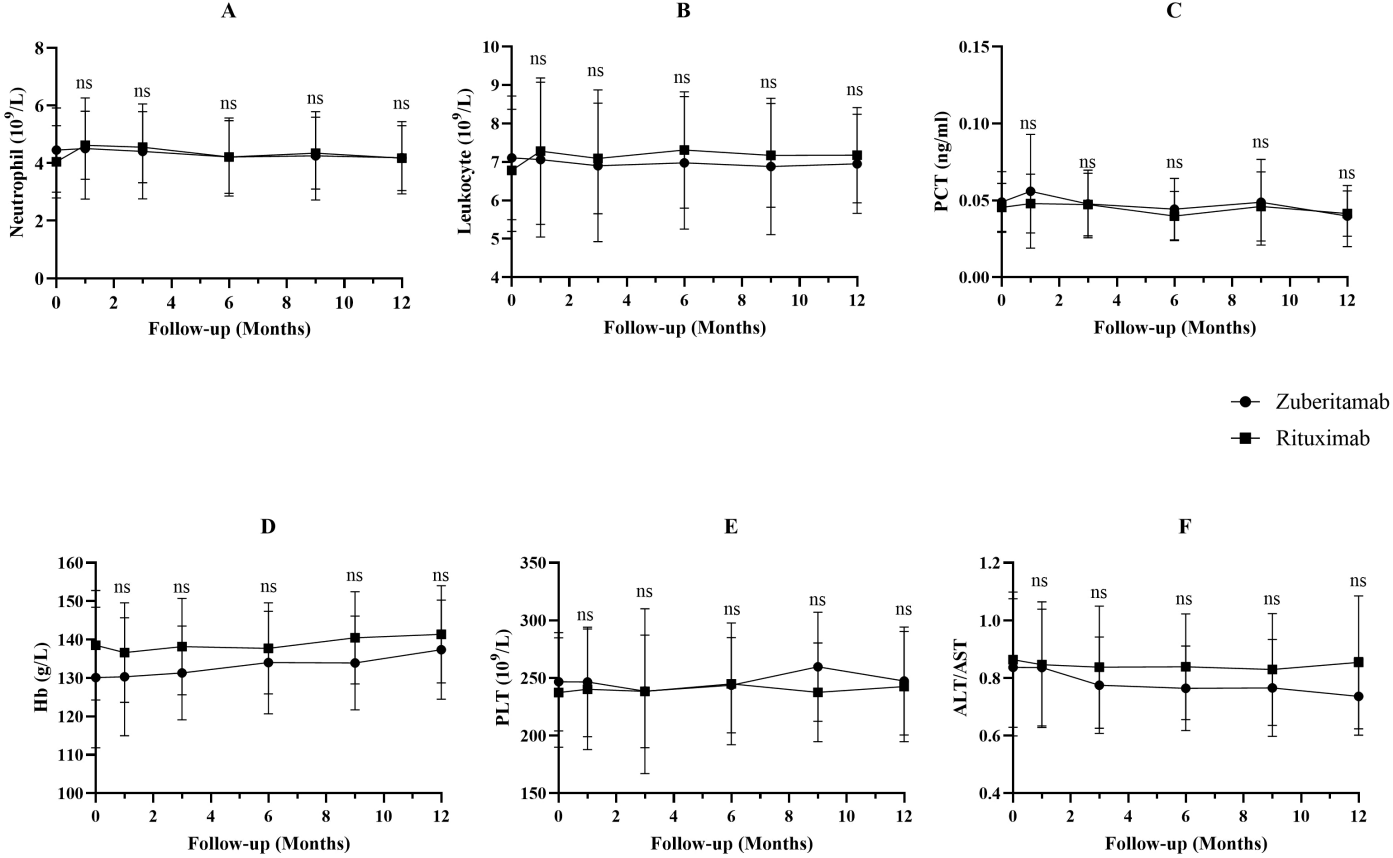
Comparison of neutrophil **(A)**, leukocyte **(B)**, PCT **(C)**, Hb **(D)**, PLT **(E)**, ALT/AST **(F)** between the zuberitamab group and rituximab group. ns P> 0.05.

Both treatment groups demonstrated comparable safety profiles. One patient (4.0%) in each group experienced CTCAE grade 1 chills and fever during antibody administration. These mild infusion-related reactions resolved with temporary infusion interruption and physical cooling, without sequelae (**Table 3**). No other prespecified safety events—including infections, neutropenia, or hepatorenal dysfunction—were reported. It is important to note that in this retrospective study, routine surveillance of serum immunoglobulin levels (IgG, IgA, IgM) was not systematically performed. Key laboratory parameters (e.g., leukocytes, neutrophils, PCT, Hb, PLT, liver enzymes) remained stable and comparable between groups (all P>0.05; **Figure 5**). The study recorded no serious adverse events, malignancies, or deaths.

**Table 3:** Summary of adverse events between the zuberitamab group and rituximab group.

Adverse Event (AE) Category	Zuberitamab (n=25)	Rituximab (n=25)	P value
Any AE	1 (4.0)	1 (4.0)	1.000
Infusion-Related Reactions	1 (4.0)	1 (4.0)	1.000
Chills and Fever (Grade 1)	1 (4.0)	1 (4.0)	1.000
Rash/Urticaria	0	0	-
Hypotension/Hypertension during infusion	0	0	-
Infectious Complications	0	0	-
Upper respiratory tract infection	0	0	-
Urinary tract infection	0	0	-
Other opportunistic infections^b^	0	0	-
Blood System Disorders	0	0	-
Leukopenia (Any Grade)	0	0	-
Neutropenia (Any Grade)	0	0	-
Anemia (Any Grade)	0	0	-
Thrombocytopenia (Any Grade)	0	0	-
Renal and Urinary Disorders	0	0	-
Acute kidney injury (AKI)	0	0	-
Worsening proteinuria	0	0	-
Elevated serum creatinine (≥1.5x baseline)	0	0	-
Hepatobiliary Disorders	0	0	-
Elevated ALT/AST (Any Grade)	0	0	-
Gastrointestinal Disorders	0	0	-
Nausea/Vomiting	0	0	-
Diarrhea	0	0	-
General Disorders and Administration Site Conditions	0	0	-
Fatigue	0	0	-
Malaise	0	0	-
Serious Adverse Events (SAEs)	0	0	-

Data are presented as n (%). AEs were graded according to CTCAE version 6.0.

b Opportunistic infections include but are not limited to: pneumonia, sepsis, etc.

## 4 Discussion

This retrospective single-center study observed high rates of clinical and immunological remission at 12 months in a cohort of PMN patients treated with zuberitamab, alongside a significant reduction in proteinuria and stable renal function. Compared to a propensity score-matched cohort treated with RTX, treatment with zuberitamab was associated with a higher rate of complete remission. The safety profile appeared favorable within the observation period. This study provides preliminary clinical evidence supporting zuberitamab as a promising therapeutic option for Chinese adults with PMN.

While current research on zuberitamab for PMN remains limited, existing data demonstrate promising efficacy. A prior IMN study reported 86% clinical remission rates (CR 19% at 3 months; 43% at 6 months) with 100% complete immunologic remission by 6 months (17). Our findings showed slightly lower 6-month remission rates (80.0% overall, 24.0% CR), potentially attributable to differences in sample size and baseline characteristics. However, this study revealed higher 12-month outcomes with 100.0% achieving clinical (80.0% CR) and immunological remission (**Table 2**). The favorable outcomes may stem from specific aspects of the study design. Requiring a complete 12-month follow-up could have selected a more adherent and clinically stable cohort, thereby enriching the study population with a favorable prognosis. This selection bias, together with the retrospective design and modest sample size, must be considered when interpreting the results.

We found zuberitamab induced profound and sustained B-cell depletion, with peripheral blood CD19^+^ B cell counts persisting below 5 cells/μL in all patients through 6-month follow-up (**Figure 2F**). This depletion effect is consistent with prior clinical observations in DLBCL (rapid reduction post-first dose maintained for ≥24 weeks) (12, 18) and idiopathic thrombocytopenic purpura (0% CD19^+^ within one week of treatment) (19). The mechanism of action of zuberitamab, like other anti-CD20 antibodies, involves ADCC, which contributes to B cell depletion and may underlie its therapeutic efficacy in immune-mediated disorders (20, 21).

This matched comparison showed that zuberitamab led to a faster proteinuria decline and a higher rate of complete clinical remission at 12 months than RTX, despite similar immunological remission and serum albumin (**Figure 4**, **Table 2**). The absence of non-responders with zuberitamab, versus one in the RTX group (consistent with known resistance (22), is intriguing but preliminary. This clinical advantage may be because zuberitamab induced deeper early B-cell depletion (**Figure 4H**), enabling a steeper proteinuria reduction that helped more patients cross the stringent threshold for complete remission. However, proteinuria is also modulated by supportive management (e.g., ACEIs/ARBs or SGLT-2 inhibitors), a potential confounder as background care was not standardized in this retrospective study. Future trials should standardize background care to clearly evaluate drug-specific effects on proteinuria.

No severe adverse events were recorded during the 12-month follow-up (**Table 3**). However, this study primarily informs us about acute infusion reactions and short-term tolerability. Its design cannot adequately assess the risks of prolonged B-cell depletion. A reported case of PJP after zuberitamab use in a different disease context highlights this potential risk (23). Importantly, the lack of systematic immunoglobulin monitoring and standardized prophylaxis against opportunistic infections limits our ability to assess long-term immunological risks, such as hypogammaglobulinemia, from profound B-cell depletion. Therefore, the favorable short-term safety profile should not be extrapolated to imply long-term immunological safety, which must be evaluated in future prospective studies with active monitoring protocols.

This study presents several key contributions to PMN treatment research. It represents the first clinical application of zuberitamab for PMN, providing a comparison with RTX regarding both efficacy and safety profiles. However, several limitations must be acknowledged. The single-center, retrospective design with a limited sample size may introduce selection bias and restrict generalizability. Importantly, the inclusion criterion requiring at least one completed treatment cycle may lead to survivorship bias. Furthermore, the zuberitamab dosing regimen was not standardized but determined through shared decision-making, introducing variability in treatment exposure that could affect outcomes. The notably high remission rates observed with zuberitamab may reflect a cohort with favorable baseline characteristics rather than fully representative real-world PMN populations. Although propensity-score matching was employed, residual confounding from unmeasured prognostic factors remains possible. The absence of adjusted multivariate models limits the ability to fully attribute outcome differences solely to the treatment effect. Furthermore, follow-up lymphocyte subset data were incomplete in some patients due to the substantial cost of testing. A *post hoc* analysis revealed no significant differences in baseline characteristics (**Table 4**). However, due to limited statistical power, this comparison cannot definitively rule out selection bias. Additionally, two patients did not undergo diagnostic kidney biopsy. While high anti-PLA_2_R antibody titers strongly suggest primary MN, the possibility of secondary MN (e.g., drug-related or malignancy-associated) or other proteinuric diseases in these unbiopsied cases may introduce bias and affect treatment response interpretation.

**Table 4:** Comparison of baseline characteristics between patients with and without missing data.

Patients (n)	Missing-data (n=6)	Complete-data (n=44)	P value
Male/Female (n)	5/1	27/17	0.399
Age (years)	50.0 (42.8-62.0)	57.0 (47.0-62.0)	0.521
Hypertension, n (%)	3 (50.0)	28 (63.6)	0.661
Diabetes mellitus, n (%)	2 (33.3)	10 (22.7)	0.621
History (months)	2.0 (1.8-16.5)	12.0 (2.0-24.0)	0.230
Baseline UPCR (g/g)	6.7 (2.6-11.9)	6.2 (4.2-8.7)	0.929
Baseline UACR (g/g)	3.5 (1.7-7.3)	3.8 (2.7-6.0)	0.788
Baseline albumin (g/L)	21.8 (18.3-39.6)	29.0 (24.6-37.4)	0.221
Baseline serum creatinine (umol/L)	81.0 (73.8-105.8)	81.5 (64.3-98.8)	0.788
Baseline eGFR (ml/min/1.73 m^2^)	90.0 (67.9-103.5)	92.7 (66.0-106.3)	0.765
Baseline PLA_2_R-Ab (RU/ml)	39.6 (2.0-144.6)	7.9 (2.0-69.4)	0.580
Baseline neutrophil (10^9^/L)	3.4 (2.6-4.4)	4.1 (3.2-5.5)	0.135
Baseline leukocyte (10^9^/L)	6.3 (5.5-7.5)	6.9 (5.9-8.3)	0.347
Baseline PCT (ng/ml)	0.049 (0.028-0.061)	0.048 (0.031-0.060)	0.976
Baseline Hb (g/L)	139.5 (124.5-149.3)	139.5 (125.0-148.5)	0.191
Baseline PLT (10^9^/L)	237.7 ± 16.0	242.7 ± 6.9	0.802
Baseline ALT/AST	0.89 ± 0.08	0.84 ± 0.04	0.617
Previous kidney biopsy, n (%)	6 (100.0)	42 (95.5)	1.000
PLA_2_R-Ab+ at the time of diagnosis,n (%)	3 (50.0)	19 (43.2)	1.000
Therapeutic schedule			
Monotherapy, n (%)	3 (50.0)	20 (45.5)	1.000
Combination therapy, n (%)	3 (50.0)	24 (54.5)	1.000
Prednisone, n (%)	2 (33.3)	7 (15.9)	0.293
Prednisone + tacrolimus, n (%)	1 (16.7)	8 (18.2)	1.000
Tacrolimus, n (%)	0 (0.0)	9 (20.5)	0.576
Zuberitamab/RTX regimen			
4×0.5g,biweekly, n (%)	5 (83.3)	23 (52.3)	0.211
2×1g,biweekly, n (%)	1 (16.7)	21 (47.7)	0.211

Conclusion: This retrospective cohort study suggests that zuberitamab may represent a viable treatment option for PMN, showing association with clinical efficacy and a favorable safety profile in this patient cohort. Treatment with zuberitamab was observed to reduce anti-PLA_2_R antibody levels and achieve sustained proteinuria reduction without compromising renal function. Furthermore, zuberitamab was associated with a higher complete remission rate compared to RTX. Large-scale, multicenter randomized controlled trials with extended follow-up are needed to fully assess zuberitamab’s long-term efficacy and safety, strengthening the evidence for its potential use in PMN.

## Data Availability

The original contributions presented in the study are included in the article/supplementary material. Further inquiries can be directed to the corresponding author.
